# Exosomes in trichology: A literature review

**DOI:** 10.1016/j.jpra.2025.11.011

**Published:** 2025-11-19

**Authors:** Kar Wai Alvin Lee, Olena Sydorchuk, Jong Keun Song, Sa Rang Lee, Eunwoo Yu, Sea Hwan Kim, Tae-Hyun Kim, Han Ah Reum Song, Seung Yong Shim, You-kyoung Cho, Han Earl Lee, Arash Jalali, Kyu-Ho Yi

**Affiliations:** a EverKeen Medical Centre, Hong Kong, Hong Kong; b SIDOR Inc., Seoul, Korea; c Pixelab Plastic Surgery Clinic, Seoul, Korea; d Department of Statistics, University of Iowa, Iowa, USA; e BBBJ Orthopedic Clinic, Seoul, Korea; f Korea National Hospital, Seoul, Korea; g Bundang CHA Medical Center, Seoul, Korea; h You and I Clinic, Seoul, Korea; i CHA University School of Medicine, Pocheon, Korea; j Opening Plastic Surgery Clinic, Korea; k One Clinic, Vancouver, Canada; l Division in Anatomy and Developmental Biology, Department of Oral Biology, Human Identificaton Research Institute, BK21 FOUR Project, Yonsei University College of Dentistry, 50-1 Yonsei-ro, Seodaemun-gu, Seoul, 03722, Korea

**Keywords:** Exosomes, Hair restoration, Trichology, Androgenetic alopecia, Alopecia areata, Hair follicle regeneration

## Abstract

**Background:**

Exosomes, nano-sized extracellular vesicles derived from various cell types, have emerged as a promising therapeutic modality in trichology. These vesicles deliver bioactive molecules, such as growth factors, cytokines, and microRNAs, that regulate key cellular processes, including proliferation, differentiation, and signaling, which are critical for hair follicle regeneration.

**Aim:**

This review synthesizes findings from preclinical and clinical studies published on the applications of exosomes for treating hair loss conditions such as androgenetic alopecia (AGA), alopecia areata (AA), and chemotherapy-induced alopecia (CIA).

**Methods:**

A comprehensive literature search was conducted using databases including MEDLINE, PubMed and Ovid databases for relevant studies published on clinical trials, diagnosis and treatment. Some papers were further reviewed using a double-blinding approach, sample size, control usage, randomization usage and objective endpoint measurements. All studies were classified according to the Oxford Center for evidence-based medicine evidence hierarchy.

**Results:**

Preclinical studies demonstrate that exosomes derived from mesenchymal stem cells (MSCs), adipose stem cells (ASCs), dermal papilla cells (DPCs) and plant sources enhance dermal papilla cell (DPC) proliferation, activate signaling pathways such as Wnt/β-catenin, VEGF, and PI3K/AKT, and promote the transition of hair follicles from the telogen to anagen phase. Clinical data, although limited, show promising results, with improvements in hair density, thickness, and follicular health. Systematic reviews and case series have highlighted the favorable safety profile of exosome-based therapies, with minimal adverse effects reported.

**Conclusion:**

Exosome therapy is a promising, minimally invasive approach for AGA, AA, and CIA, supported by robust preclinical biology but preliminary and heterogeneous clinical data. Progress to routine practice will require adequately powered, multicenter randomized trials with standardized outcomes, along with harmonized manufacturing and regulatory frameworks (GMP-compliant production, batch-level quality control) to ensure reproducibility and safety.

## Introduction

Hair loss disorders—including androgenetic alopecia (AGA), alopecia areata (AA), and chemotherapy-induced alopecia (CIA)—are common and psychologically burdensome conditions.^1^, ^2^, ^3^ Standard options such as topical minoxidil, oral finasteride, platelet-rich plasma (PRP), and hair transplantation offer variable efficacy, durability, and tolerability,^4^, ^5^, ^6^, ^7^, ^8^ prompting interest in minimally invasive alternatives.

Exosomes—nano-sized extracellular vesicles that mediate intercellular communication—carry proteins, cytokines, growth factors, lipids, and nucleic acids that modulate repair pathways in skin and hair.^9^, ^10^, ^11^ In trichology, exosomes stimulate dermal papilla cell activity, angiogenesis, and follicle cycling,^12^ engaging Wnt/β-catenin, VEGF, and PI3K/AKT signaling^13^, ^14^, ^15^ and counteracting dihydrotestosterone-mediated inhibition.^16^ Early clinical reports show improvements in hair density and thickness with favorable safety, and combination approaches (e.g., with low-level laser therapy) may augment outcomes.^17^^,^^18^

This review synthesizes preclinical and clinical evidence on exosome-based therapies for alopecia, outlines mechanistic underpinnings, and highlights limitations in isolation, characterization, and delivery protocols that currently impede standardization—thereby defining priorities for future trials and clinical translation.

## Materials and methods

### Study design and scope

This work is a narrative review that incorporated structured database searches and qualitative synthesis. We summarized preclinical (in vitro/in vivo) and clinical evidence on exosome-based therapies for hair loss (androgenetic alopecia, alopecia areata, chemotherapy-induced alopecia), with results presented in separate subsections to preserve the evidence hierarchy. No quantitative meta-analysis was performed.

### Data sources and search strategy

Searches were conducted in MEDLINE (via PubMed), Embase (via Ovid), Web of Science Core Collection, and Cochrane CENTRAL, from database inception to 31 August 2025. We used controlled vocabulary (MeSH/Emtree) and free-text terms combining exosome concepts with trichology terms:

(“exosome*” OR “extracellular vesicle*” OR “small extracellular vesicle*” OR “sEV*”) AND (hair OR alopecia OR trichology OR “dermal papilla”).

Reference lists of included articles and relevant reviews were hand-searched to identify additional records.

### Eligibility criteria

We included original preclinical studies (cellular/animal) and clinical studies (randomized or non-randomized trials, prospective/retrospective cohorts, case series, case reports) evaluating exosome-based or exosome-enriched small extracellular vesicle interventions for hair disorders with efficacy and/or safety outcomes. Exclusion criteria were: non-hair indications, non-original articles (narrative reviews, editorials, letters without primary data), conference abstracts without sufficient data, duplicate publications, and studies lacking exosome-related interventions. When studies used differing terminology (e.g., “exosomes,” “EVs,” “sEVs”), we retained the authors’ wording but treated them under the umbrella of exosome-enriched small EV preparations for synthesis.

### Screening and selection

Two reviewers independently screened titles/abstracts and then full texts for eligibility; disagreements were resolved by discussion.

### Data extraction

From each eligible study we extracted: study design, population and alopecia subtype, sample size, exosome source (e.g., MSC/ADSC/DPC, platelet-derived), isolation/characterization methods (when reported), dose and delivery (injection/topical; schedule), comparator (if any), follow-up duration, primary efficacy endpoints (e.g., hair density/thickness, anagen/telogen ratio), key mechanistic readouts (e.g., Wnt/β-catenin, PI3K/AKT, VEGF), and safety/adverse events. Clinical studies were summarized in a descriptive table (see Table 1).

**Table 1:** Summary of clinical studies on exosome-based therapies for hair loss.

Study (Year)	Design/Oxford level	N (Patients)	Condition	Exosome source	Delivery method	Follow-up	Key outcomes	Adverse events
Wan et al., 2023^19^	Prospective cohort (IIb)	72	AGA	ADSC-derived	Scalp injection	6 mo	↑Hair density & thickness; improved follicular cycling	Mild erythema
Park et al., 2024^20^	Retrospective (IV)	39	AGA / AA	ADSC-derived	Injection	6 mo	↑ Density & thickness	None
Amini et al., 2024^21^	Randomized controlled (Ib)	40	AGA	Plant-derived exosomes	Topical	24 wk	↑ Hair density vs placebo	None
Lee et al., 2024^22^	Prospective (IIb)	58	AGA	ADSC-derived	Injection	6 mo	↑ Density, thickness, satisfaction	None
Ersan et al., 2023^23^	Prospective (IIIc)	26	AGA	MSC-derived	Injection	6 mo	↑ Density, thickness	None
								None
Norooznezhad et al., 2023^24^	Case report (IV)	1	CIA	MSC-derived EV-enriched	Injection	6 mo	Hair regrowth, restored coverage	None
Hassan et al., 2022^25^	Case series (IV)	12	AGA	MSC-derived	Injection vs PRP	3–6 mo	Exosome > PRP for density & durability	Mild edema
Mirzadeh et al., 2024^18^	Case series (IV)	10	AGA	Autologous plasma-derived	Injection + LLLT	12 wk	Synergistic improvement vs single modality	None
Chen et al., 2023^26^	Case series (IV)	5	ATN	MSC-derived	Injection	12 wk	↑ Hair strength & texture	None

Abbreviations: AGA, androgenetic alopecia; AA, alopecia areata; CIA, chemotherapy-induced alopecia; ATN, acquired trichorrhexis nodosa; MSC, mesenchymal stem cell; ADSC, adipose-derived stem cell; PRP, platelet-rich plasma; LLLT, low-level laser therapy; EV, extracellular vesicle.

### Appraisal and evidence hierarchy

We qualitatively appraised design features relevant to internal validity (randomization, blinding, control use, objective endpoint assessment, sample size) and classified each clinical study using the Oxford Centre for Evidence-Based Medicine levels of evidence. Preclinical and clinical evidence are synthesized separately to maintain hierarchy and avoid conflation.

### Language and publication restrictions

Searches were limited to English-language publications. A total of ⟨N_nonEnglish_excluded⟩ non-English records were identified but excluded due to feasibility constraints; their titles/abstracts did not alter the thematic conclusions.

### Synthesis approach

Given heterogeneity in exosome sources, isolation/characterization methods, dosing, delivery routes, endpoints, and follow-up, we performed a qualitative, narrative synthesis without pooled effect estimates. Where feasible, we highlight convergent mechanistic pathways and summarize clinical outcomes descriptively.

### Protocol and ethics

As a narrative review of published data, this study was not registered and did not require institutional ethics approval.

## Result

A total of 734 records were retrieved, of which 328 were screened and 42 studies were included for qualitative synthesis. To maintain evidence hierarchy and readability, we summarize individual studies with their design and Oxford level; a descriptive summary of clinical studies (study design, sample size, exosome source, delivery, follow-up, outcomes, adverse events, Oxford level) is provided in Table 1.

Queen et al.^27^ review clinical studies on the use of exosomes for treating hair loss, particularly androgenetic alopecia and alopecia areata. It summarizes evidence from recent clinical studies, noting that most trials report positive outcomes with minimal adverse effects. However, the authors emphasize significant gaps, such as small sample sizes, short follow-up periods, and variability in exosome preparation methods. They also discuss the lack of standardization in treatment protocols and the need for long-term safety data. The authors conclude that while exosome therapy is a promising treatment for hair loss, more rigorous, large-scale randomized controlled trials are necessary to validate its efficacy (Level IIIa).

Wan and colleagues^19^ conducted a prospective study evaluating the efficacy of exosome therapy for androgenetic alopecia. In this study, 72 patients underwent scalp injections of exosomes derived from adipose-derived stem cells. Over a 6-month follow-up period, significant improvements in hair density, thickness, and scalp coverage were observed, as measured through trichoscopic analysis and standardized photographs. Patient-reported outcomes also indicated high satisfaction with the treatment. The authors proposed that exosomes act by delivering growth factors and signaling molecules, such as VEGF and Wnt proteins, to hair follicles, enhancing follicular regeneration and reducing miniaturization. Adverse effects were minimal, limited to mild erythema and transient discomfort at the injection sites. This study supports exosome therapy as a safe and effective non-surgical option for AGA management (Level IIb).

Schaffer et al.^28^ conducted a scoping review to evaluate the applications of exosome-based therapies for hair loss across various conditions, including androgenetic alopecia, alopecia areata, and chemotherapy-induced alopecia. The review synthesized findings from preclinical and clinical studies, highlighting the mechanisms through which exosomes derived from sources such as adipose-derived stem cells, dermal papilla cells, and platelet-rich plasma exert their effects. These mechanisms include enhancing dermal papilla cell proliferation, modulating immune responses, reducing oxidative stress, and activating key pathways like Wnt/β-catenin and VEGF signaling. The review also addressed challenges in exosome delivery, such as optimizing dosing, injection protocols, and scalability for clinical use. Overall, the authors concluded that exosome therapy holds significant promise as a non-invasive, regenerative treatment for hair loss, though further large-scale, randomized clinical trials are needed to establish safety, efficacy, and standardized protocols (Level Ia).

Chen and colleagues^26^ presented a case series evaluating the efficacy of mesenchymal stem cell (MSC) exosome therapy for patients with acquired trichorrhexis nodosa (ATN), a hair shaft disorder characterized by nodular fragility and breakage. The study included five patients who underwent multiple scalp injections of MSC-derived exosomes over a 12-week period. The treatment led to significant improvements, including reduced hair fragility, increased hair strength, and improved texture. Microscopic examination revealed restoration of the hair cuticle and reduced nodular damage. The therapeutic benefits were attributed to the regenerative properties of MSC exosomes, which are rich in growth factors, microRNAs, and anti-inflammatory molecules that promote hair shaft repair and scalp health. No adverse effects were reported, and patient satisfaction scores were high, suggesting MSC exosome therapy as a safe and effective option for ATN (Level IV).

Amini and coworkers^21^ conducted a pilot randomized controlled trial to evaluate the efficacy of a novel exosome-containing plant extract formulation for treating male androgenetic alopecia (AGA). The study involved 40 male participants with mild to moderate AGA, randomly assigned to either the exosome-containing plant extract group or the placebo group. Participants applied the topical formulation daily for 24 weeks. The results demonstrated that the exosome group showed significant improvements in hair density, thickness, and scalp coverage compared to the placebo group. Trichoscopic analysis revealed increased follicular activity and reduced miniaturization in the exosome group. No adverse effects were reported, and the participants rated the treatment as well-tolerated. The authors concluded that plant-derived exosomes could serve as an effective and safe topical therapy for male AGA, though larger trials are necessary to confirm these findings and optimize dosing (Level Ib).

Chu et al.^29^ investigated the role of exosome-derived long non-coding RNA (lncRNA) AC010789.1 in promoting the growth of hair follicle stem cells (HFSCs) and counteracting androgenetic alopecia (AGA). The study revealed that exosomes from dermal papilla cells (DPCs) contained AC010789.1, a lncRNA modified by the RNA demethylase FTO and RNA-binding protein hnRNPA2B1. This modified lncRNA was shown to activate the S100A8/Wnt/β-catenin signaling pathway, which plays a key role in HFSC proliferation, migration, and differentiation. In vitro experiments demonstrated that exosomes enriched with AC010789.1 significantly enhanced HFSC activity, while in vivo studies in AGA mouse models showed that treatment with these exosomes resulted in accelerated hair regrowth, increased follicle density, and reduced follicular miniaturization. The findings suggest that targeting lncRNA modifications in exosomes offers a novel therapeutic strategy for treating AGA (Level V).

Fu and colleagues^30^ explored the therapeutic effects of mesenchymal stem cell (MSC)-derived exosomes on androgenetic alopecia. The study demonstrated that MSC exosomes significantly enhanced hair follicle development and mitigated the effects of AGA by promoting dermal papilla cell proliferation, migration, and extracellular matrix deposition. Mechanistically, the exosomes were found to activate key signaling pathways, including Wnt/β-catenin, VEGF, and FGF, which are crucial for hair follicle regeneration and vascularization. In a mouse model of AGA, MSC exosome treatment led to increased hair density, thicker hair shafts, and improved follicular structure compared to controls. The researchers concluded that MSC exosomes represent a promising, minimally invasive therapeutic option for treating AGA and restoring hair growth, though further clinical studies are necessary to validate these findings and optimize treatment protocols (Level V).

Lu and coworkers^31^ investigated the effects of platelet-rich plasma (PRP)-derived exosomes on hair follicle growth, with a focus on the Wnt/β-catenin signaling pathway. The study demonstrated that PRP-derived exosomes significantly promoted the proliferation, migration, and differentiation of dermal papilla cells (DPCs) in vitro. Mechanistically, the exosomes were found to activate the Wnt/β-catenin pathway, which is critical for hair follicle development and cycling. In a mouse model, treatment with PRP-derived exosomes resulted in accelerated hair regrowth, increased follicle density, and a transition of hair follicles from the telogen to anagen phase. The study also observed enhanced vascularization around the hair follicles, supporting follicular health and growth. The authors concluded that PRP-derived exosomes represent a promising, non-invasive therapeutic approach for promoting hair regrowth, offering an alternative to traditional PRP therapy with potentially enhanced efficacy (Level V).

Chen et al.^31^ investigated the effects of exosomes derived from human umbilical cord mesenchymal stem cells (hUC-MSCs) on the growth and function of human hair dermal papilla cells (hDPCs). The study demonstrated that hUC-MSC-derived exosomes significantly enhanced hDPC proliferation, migration, and extracellular matrix production. Mechanistic analysis revealed that the exosomes activated the Wnt/β-catenin and PI3K/AKT signaling pathways, both critical for hair follicle development and hair growth. In addition, the exosomes were found to deliver growth factors and microRNAs that modulated the expression of genes associated with the anagen (growth) phase of the hair follicle cycle. In vitro experiments confirmed that treating hDPCs with these exosomes improved their ability to sustain hair follicle growth. The authors concluded that hUC-MSC-derived exosomes have strong potential as a therapeutic option for promoting hair regrowth and treating hair loss conditions such as androgenetic alopecia (AGA), pending further clinical trials (Level V).

Ersan and colleagues^23^ conducted a prospective study evaluating the effectiveness of exosome therapy in treating androgenetic alopecia. Participants treated with exosomes showed significant improvement in hair density, thickness, and overall scalp health compared to baseline. The study also reported minimal adverse effects, suggesting a favorable safety profile. While the results are promising, the authors acknowledge limitations such as the small sample size and lack of a control group. They emphasize the need for further randomized controlled trials to confirm these findings and establish standardized protocols for exosome preparation and administration (Level IIIc).

Lee and coworkers^22^ investigated the efficacy of adipose stem cell-derived exosomes (ADSC-exosomes) in hair regeneration through both preclinical and clinical studies. Preclinical experiments demonstrated that ADSC-exosomes enhance dermal papilla cell proliferation, stimulate hair follicle growth, and upregulate essential signaling pathways, such as Wnt/β-catenin. In the clinical phase, 58 patients with androgenetic alopecia underwent scalp injections of ADSC-exosomes, showing significant improvements in hair density, thickness, and growth rate. Results were assessed using trichoscopy, patient satisfaction surveys, and blinded dermatological evaluations. The study emphasized the exosomes’ ability to deliver growth factors and cytokines directly to the follicle microenvironment, promoting follicular regeneration. No severe adverse effects were reported, indicating a favorable safety profile. These findings suggest that ADSC-exosomes are a promising, non-invasive therapeutic option for hair restoration (Level IIb).

Norouzi et al.^32^ reviewed the therapeutic potential of stem cell-derived exosomes for skin rejuvenation and hair regrowth. For hair regrowth, exosomes derived from mesenchymal stem cells (MSCs), adipose-derived stem cells (ADSCs), and dermal papilla cells were shown to activate signaling pathways like Wnt/β-catenin, VEGF, and FGF, which are crucial for hair follicle regeneration and growth. Preclinical and early clinical studies cited in the review demonstrated improved hair density, follicle size, and scalp vascularization with exosome therapy. The authors also discussed the advantages of exosomes over traditional stem cell therapies, including higher safety profiles, scalability, and ease of delivery. Challenges such as standardizing exosome isolation, dosing, and application protocols were noted as areas requiring further research (Level Ia).

Hassan and colleagues^25^ conducted a case series study comparing the efficacy of platelet-rich plasma (PRP) and exosome-based therapies for hair loss in 12 patients with androgenetic alopecia (AGA). The participants were divided into two groups: six received PRP injections, and six were treated with exosome injections derived from mesenchymal stem cells (MSCs). Both groups underwent four treatment sessions over 3 months. The study found that both PRP and exosome therapies significantly improved hair density and thickness, but the exosome group demonstrated superior results in terms of hair regrowth speed, follicle density, and overall patient satisfaction. Exosome therapy was also associated with longer-lasting effects, as follow-ups at 6 months showed sustained improvements in the exosome group compared to a slight decline in the PRP group. The authors attributed the superior performance of exosomes to their bioactive cargo, including growth factors, cytokines, and microRNAs, which target multiple pathways involved in hair follicle regeneration (Level IV).

Zhou and coworkers^33^ reviewed the applications of exosomes in hair growth and regeneration, focusing on their mechanisms of action, therapeutic potential, and challenges. The paper discussed exosomes derived from various cell types, such as mesenchymal stem cells (MSCs), dermal papilla cells (DPCs), and adipose-derived stem cells (ADSCs), highlighting their ability to promote hair follicle development, enhance hair shaft growth, and stimulate stem cell activity. These effects are mediated through bioactive molecules carried by exosomes, including growth factors, cytokines, and microRNAs that regulate key pathways such as Wnt/β-catenin, FGF, and VEGF signaling. Preclinical and clinical studies reviewed in the article demonstrated promising results in improving hair density and thickness in individuals with androgenetic alopecia and alopecia areata. The authors also addressed challenges, including optimizing delivery systems, scaling production, and standardizing protocols. They concluded that exosome therapy represents a cutting-edge approach for hair loss treatment, though further research is needed to address existing limitations and ensure widespread clinical adoption (Level Ia).

Mao et al.^34^ investigated the effects of exosomes derived from umbilical cord mesenchymal stem cells (UC-MSCs) on hair regrowth, focusing on the RAS/ERK signaling pathway. The study demonstrated that UC-MSC-derived exosomes significantly enhanced hair follicle regeneration and hair growth in C57BL/6 mice. Mechanistically, the exosomes were shown to upregulate the RAS/ERK pathway, which promotes dermal papilla cell (DPC) proliferation, migration, and secretion of growth factors essential for hair follicle development. In vivo experiments revealed that mice treated with UC-MSC exosomes exhibited increased hair density, thicker hair shafts, and improved follicular cycling compared to untreated controls. Additionally, the treatment was well-tolerated, with no observed adverse effects. The authors concluded that UC-MSC exosomes represent a promising therapeutic approach for promoting hair regrowth, with the RAS/ERK pathway playing a key role in their mechanism of action (Level V).

Hu and colleagues^35^ examined the effects of exosomes derived from umbilical mesenchymal stem cells (uMSCs) on hair regrowth in alopecia areata (AA). The study found that uMSC-derived exosomes enhanced the proliferation, migration, and wound-healing activities of human hair follicular keratinocytes (HHFKs) in vitro. Mechanistically, the exosomes were shown to deliver growth factors and microRNAs that activated key signaling pathways, including Wnt/β-catenin and PI3K/AKT, which are critical for hair follicle regeneration and keratinocyte function. In a mouse model of AA, treatment with uMSC exosomes significantly improved hair regrowth, increased follicle density, and reduced inflammatory infiltration around hair follicles. The findings suggest that uMSC-derived exosomes accelerate keratinocyte-driven processes to restore normal hair follicle cycling and promote regrowth in AA. The authors concluded that this exosome-based approach is a promising therapeutic strategy for AA, with potential for clinical translation pending further studies (Level V).

Mirzadeh and coworkers^18^ reported on a case series examining the combined effects of low-level laser therapy (LLLT) and autologous exosome therapy on hair growth in patients with androgenetic alopecia (AGA). The study included 10 patients who underwent a treatment regimen combining LLLT sessions and scalp injections of exosomes derived from their autologous plasma. The results showed significant improvements in hair density, thickness, and scalp coverage after 12 weeks of treatment. Trichoscopic analysis revealed reduced follicular miniaturization and increased anagen-to-telogen hair ratio. Patients reported high satisfaction with the therapy, and no adverse effects were observed. The authors proposed that the combination of LLLT and exosome therapy acts synergistically, with LLLT enhancing the bioactivity and delivery of exosomes to hair follicles. They concluded that this dual approach is a promising, minimally invasive strategy for AGA management, warranting further investigation in larger, controlled clinical trials (Level IV).

Cheng et al.^36^ reviewed the roles of exosomes in regulating hair follicle growth, focusing on their mechanisms of action, therapeutic potential, and applications in hair loss conditions such as androgenetic alopecia (AGA) and alopecia areata (AA). The review highlighted that exosomes, derived from sources such as mesenchymal stem cells (MSCs), dermal papilla cells (DPCs), and adipose stem cells (ASCs), carry bioactive molecules like proteins, lipids, and nucleic acids (e.g., microRNAs and lncRNAs). These molecules were shown to influence key signaling pathways, including Wnt/β-catenin, VEGF, and TGF-β/SMAD3, to promote follicle growth, anagen phase induction, and angiogenesis. The authors summarized preclinical and clinical studies demonstrating the efficacy of exosome therapy in enhancing hair density, thickness, and scalp health. Additionally, the review discussed challenges such as the scalability of exosome production, delivery methods (e.g., topical application, injections), and safety concerns. The authors concluded that exosomes represent a promising, cell-free approach to hair follicle regeneration, offering potential alternatives to traditional therapies, with further research needed to optimize protocols and confirm long-term efficacy (Level Ia).

Gupta and colleagues^17^ explores the emerging role of exosome-based therapies in hair restoration, focusing on their efficacy, safety, and potential future applications. Preliminary evidence from small-scale studies suggests that exosome treatments may improve hair density and thickness, particularly in androgenetic alopecia and alopecia areata. However, the lack of standardized protocols, variability in exosome sources, and limited high-quality clinical trials temper the enthusiasm. Safety profiles appear favorable, with minimal adverse effects reported. The authors emphasize the need for larger randomized controlled trials to establish efficacy, dosing guidelines, and long-term safety (Level 3b).

Wang and coworkers^37^ investigated the use of exosomes secreted by hair papilla cells (HPC-exosomes) for the treatment of androgenetic alopecia (AGA) and studied the intervention effect of lactotransferrin (LTF) on exosome activity. The study revealed that HPC-exosomes promote dermal papilla cell proliferation, enhance hair follicle development, and activate the Wnt/β-catenin signaling pathway. Additionally, LTF, a bioactive protein known for its anti-inflammatory and regulatory properties, was shown to enhance the regenerative capacity of HPC-exosomes by increasing the expression of hair growth-promoting factors. In vivo studies using mouse models demonstrated significant improvement in hair density, follicle size, and growth rate in exosome-treated groups, with further enhancement observed when combined with LTF. These findings suggest that HPC-exosomes, particularly when augmented by LTF, offer a promising therapeutic option for AGA (Level V).

Norooznezhad and coworkers^24^ reported a case of persistent chemotherapy-induced alopecia (CIA) successfully treated with exosome-enriched extracellular vesicles (EVs) derived from human mesenchymal stromal cells (MSCs). The patient, who experienced prolonged hair loss after chemotherapy, was treated with multiple scalp injections of MSC-derived exosome-enriched EVs over a 6-month period. The therapy resulted in significant hair regrowth, improved hair density, and restoration of scalp coverage. The authors attributed these results to the regenerative properties of MSC-derived exosomes, which deliver bioactive molecules such as growth factors, cytokines, and microRNAs, enhancing hair follicle health, reducing inflammation, and promoting vascularization. The treatment was well-tolerated, with no reported adverse effects. This case highlights the potential of MSC-derived exosome therapy as a novel and effective treatment for persistent CIA (Level IV).

**Figure 1:**
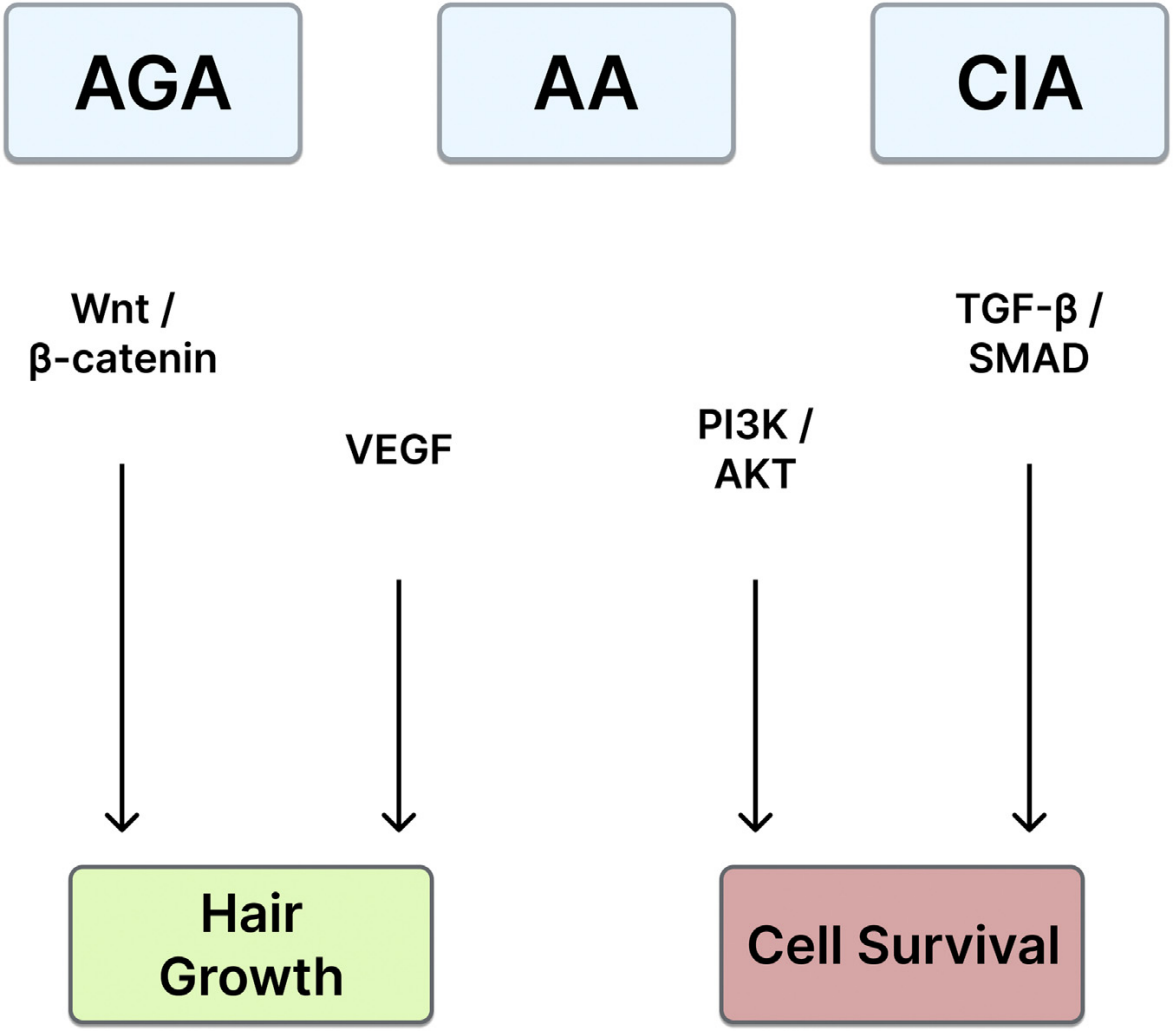
Schematic summary of key signaling pathways involved in exosome-mediated hair follicle regeneration across androgenetic alopecia (AGA), alopecia areata (AA), and chemotherapy-induced alopecia (CIA). Exosomes activate Wnt/β-catenin, VEGF, and PI3K/AKT signaling to promote hair follicle proliferation, angiogenesis, and survival, while modulating TGF-β/SMAD pathways to counteract apoptosis and follicular regression.

Gupta et al.^38^ reviewed the emerging role of exosomes in hair restoration, summarizing preclinical and clinical studies, as well as their mechanisms of action. The review highlighted that exosomes, derived from sources such as mesenchymal stem cells (MSCs), dermal papilla cells (DPCs), and adipose-derived stem cells (ADSCs), promote hair follicle regeneration by delivering bioactive molecules, including proteins, lipids, and microRNAs. These molecules were shown to stimulate key signaling pathways, such as Wnt/β-catenin, VEGF, and FGF, which are critical for follicular development, angiogenesis, and hair growth. Clinical studies demonstrated that exosome therapy improves hair density, thickness, and scalp health with minimal side effects. The authors also discussed practical considerations, including dosing strategies, delivery methods (e.g., topical applications and injections), and challenges such as scalability and cost. They concluded that exosome therapy offers a novel, minimally invasive approach to hair restoration, though more large-scale, randomized trials are needed to standardize treatment protocols and confirm long-term efficacy (Level Ia) (Figure 1).

Liang et al.^16^ investigated the therapeutic effects of adipose mesenchymal stromal cell (AMSC)-derived exosomes carrying miR-122-5p on hair follicle growth under the inhibitory influence of dihydrotestosterone (DHT), a key factor in androgenetic alopecia (AGA). The study revealed that miR-122-5p, delivered via AMSC-derived exosomes, counteracted the suppressive effects of DHT on dermal papilla cells (DPCs). Mechanistically, miR-122-5p inhibited the TGF-β1/SMAD3 signaling pathway, which is known to mediate hair follicle miniaturization and promote catagen (regression) phase induction. In vitro experiments demonstrated that these exosomes restored DPC proliferation, migration, and extracellular matrix production in the presence of DHT. In a mouse model of AGA, topical application of AMSC-derived exosomes significantly improved hair density, follicular size, and regrowth rates compared to untreated controls. The authors concluded that miR-122-5p-loaded exosomes represent a novel therapeutic strategy for treating DHT-induced hair loss, with the potential to reverse AGA progression by targeting TGF-β1/SMAD3 signaling (Level V).

Kost and colleagues^39^ review the application of exosome therapy in hair regeneration, focusing on its mechanisms, current evidence, and challenges. The review discusses studies demonstrating promising improvements in hair density and scalp health, particularly in androgenetic alopecia. Despite encouraging results, the authors emphasize significant challenges, such as the lack of standardized protocols, variability in exosome isolation and characterization, and the absence of large-scale randomized controlled trials. They also address potential safety concerns, including immune responses and long-term effects. The authors conclude that while exosome therapy shows potential, more rigorous studies are needed to establish its efficacy and safety (Level IIIb).

Park and coworkers^20^ explored the effects of exosomes derived from adipose-derived stem cells (ADSCs) on hair loss through a retrospective analysis of 39 patients. Exosomes, nanosized extracellular vesicles involved in cell communication, are emerging as potential therapeutic agents in trichology due to their regenerative properties. In this study, the exosomes were injected into the scalp of patients diagnosed with androgenetic alopecia or alopecia areata. Clinical outcomes, measured via standardized photographs and patient self-assessment, showed significant improvement in hair density and thickness after treatment. The authors highlighted exosomes’ role in promoting hair follicle growth by delivering bioactive molecules such as growth factors and cytokines to dormant follicles. The treatment was well-tolerated, with minimal side effects reported. These findings support the potential of ADSC-derived exosomes as a novel, minimally invasive therapy for hair restoration (Level IV).

Li et al.^40^ examined the therapeutic potential of exosomes derived from adipose-derived stem cells (ADSCs) for treating immune-mediated alopecia, such as alopecia areata. The study focused on the immunomodulatory properties of ADSC-exosomes, which can regulate immune responses by delivering bioactive molecules, including cytokines, proteins, and microRNAs. In preclinical models, ADSC-exosomes reduced inflammatory infiltration around hair follicles and restored normal hair growth by modulating T-cell activity, particularly reducing the overactivation of CD8+ cytotoxic T cells and promoting regulatory T cell function. The study also highlighted the ability of ADSC-exosomes to enhance the hair follicle microenvironment by promoting follicular stem cell activation. These findings suggest that ADSC-exosomes could serve as a non-invasive, immune-modulating therapy for immune-mediated alopecia, providing a dual benefit of reducing inflammation and stimulating hair regeneration (Level V).

Zhang and colleagues^41^ investigated the effects of photobiomodulation (PBM) on hair regeneration in injured skin, focusing on its impact on dermal papilla cells (DPCs). The study showed that PBM, using low-level laser therapy (LLLT), enhanced the migration and exosome secretion of DPCs in vitro. These exosomes were found to carry bioactive molecules that activated key signaling pathways, including Wnt/β-catenin and VEGF, which are essential for hair follicle regeneration and angiogenesis. In a mouse model of injured skin, PBM treatment significantly accelerated wound healing, increased hair follicle density, and promoted hair regrowth. The findings suggest that PBM enhances the regenerative capacity of DPCs by stimulating exosome production, making it a promising non-invasive approach for hair regeneration in both injured skin and hair loss conditions (Level V).

Kim and coworkers^42^ explored the potential of colostrum-derived exosomes for promoting hair regeneration, focusing on their ability to induce the transition of hair follicles from the telogen (resting) phase to the anagen (active growth) phase. The study found that colostrum-derived exosomes are rich in bioactive molecules, such as growth factors and microRNAs, which stimulate dermal papilla cells (DPCs) and hair follicular keratinocytes. These exosomes were shown to activate key signaling pathways, including Wnt/β-catenin and PI3K/AKT, which are essential for initiating and sustaining the anagen phase. In a mouse model, topical application of colostrum-derived exosomes significantly accelerated hair regrowth, increased follicle density, and improved hair shaft thickness. The treatment was well-tolerated, with no observed adverse effects. The authors concluded that colostrum-derived exosomes represent a natural, non-invasive therapeutic option for hair regeneration, though further studies are required to evaluate their efficacy and scalability for clinical use (Level V).

Wu et al.^43^ explored the role of adipose-derived stem cell (ADSC) exosomes in promoting hair regeneration through preclinical studies. The study demonstrated that ADSC exosomes enhance the proliferation and migration of dermal papilla cells and activate key signaling pathways, including Wnt/β-catenin and Akt, which are crucial for hair follicle development and growth. In vivo experiments on mouse models showed that exosome treatment resulted in increased hair density, follicle size, and growth rate compared to controls. The authors also observed reduced apoptosis and improved vascularization in the treated areas, contributing to a healthier follicular microenvironment. These findings suggest that ADSC exosomes are a promising therapeutic option for hair loss conditions, offering a non-invasive approach to stimulate hair follicle regeneration (Level V).

Ogawa and colleagues^44^ investigated the effects of exosomes derived from fisetin-treated keratinocytes on hair growth. Fisetin, a plant-derived flavonoid known for its anti-inflammatory and antioxidant properties, was used to stimulate keratinocytes, which subsequently secreted bioactive exosomes. These exosomes were found to promote dermal papilla cell proliferation, enhance the expression of hair growth-related genes (e.g., Wnt/β-catenin signaling components), and improve vascularization in hair follicles. In vivo experiments on mice demonstrated accelerated hair regrowth in exosome-treated areas compared to controls. The study highlighted the dual benefits of fisetin’s protective effects on keratinocytes and the regenerative potential of their exosomes. These findings suggest that fisetin-treated keratinocyte-derived exosomes could serve as a novel, non-invasive therapy for hair loss, leveraging both natural compounds and exosome-based delivery mechanisms (Level V).

Nilforoushzadeh and coworkers^45^ evaluated the effects of exosomes derived from adipose-derived stem cells (ADSCs) and platelet-rich plasma (PRP) on the inductivity of hair dermal papilla cells (DPCs). The study demonstrated that both ADSC- and PRP-derived exosomes enhanced the proliferation and inductive potential of DPCs in vitro. These exosomes upregulated the expression of hair growth-related genes, including Wnt/β-catenin pathway components, VEGF, and FGF-7, which are crucial for follicle development and angiogenesis. The combination of ADSC and PRP exosomes showed a synergistic effect, significantly enhancing DPC function more than either exosome type alone. The findings suggest that ADSC- and PRP-derived exosomes can create a supportive microenvironment for hair follicle regeneration and might serve as a combined therapeutic approach for hair loss (Level V).

Ajit et al.^46^ reviewed the therapeutic potential of mesenchymal stem cell (MSC)-derived exosomes for the treatment of alopecia. The article highlighted the biological properties of MSC exosomes, including their ability to deliver bioactive molecules such as growth factors, cytokines, and microRNAs, which modulate signaling pathways involved in hair follicle regeneration. The review discussed in vitro and in vivo studies showing that MSC-derived exosomes promote dermal papilla cell (DPC) proliferation, enhance follicle size, stimulate angiogenesis, and improve the overall health of hair follicles. Key signaling pathways such as Wnt/β-catenin, VEGF, and PI3K/AKT were identified as central to the regenerative effects of exosomes. The authors also addressed challenges related to scaling production, optimizing delivery systems, and standardizing treatment protocols. They concluded that MSC exosomes hold significant promise as a minimally invasive, cell-free therapeutic strategy for alopecia, though further research is needed to validate their efficacy in clinical settings (Level Ia).

Hu and colleagues^47^ investigated the role of dermal exosomes enriched with miR-218-5p in promoting hair regeneration through the regulation of β-catenin signaling. The study demonstrated that exosomes isolated from dermal cells significantly enhanced hair follicle growth and keratinocyte proliferation in vitro. Mechanistically, miR-218-5p, a microRNA highly expressed in these exosomes, was found to activate β-catenin signaling by inhibiting the expression of SFRP2, a Wnt pathway antagonist. In vivo experiments in mice revealed that treatment with dermal exosomes containing miR-218-5p led to accelerated hair regrowth, increased follicle density, and enhanced vascularization in treated areas. The findings highlight the potential of exosome-based delivery of miR-218-5p as a targeted, non-invasive therapeutic strategy for hair loss (Level V).

Kwack and coworkers^48^ investigated the effects of exosomes derived from human dermal papilla cells (hDPC-exosomes) on hair growth in cultured human hair follicles and dermal papilla spheres. The study demonstrated that hDPC-exosomes significantly promoted hair elongation and keratinocyte proliferation in cultured human hair follicles. Additionally, they augmented the hair-inductive capacity of dermal papilla spheres by upregulating key signaling pathways, including Wnt/β-catenin and fibroblast growth factor (FGF) signaling, which are vital for hair follicle development and cycling. These findings suggest that hDPC-exosomes can serve as a potent agent for enhancing hair follicle regeneration and may be a promising therapeutic option for treating hair loss conditions (Level V).

Zhou et al.^49^ investigated the role of exosomes derived from dermal papilla cells (DPC-exosomes) in regulating hair follicle development. The study demonstrated that DPC-exosomes promote the proliferation, migration, and differentiation of human outer root sheath cells (ORSCs) in vitro. Mechanistically, the exosomes were found to activate the Wnt/β-catenin signaling pathway, which is essential for hair follicle morphogenesis and cycling. In vivo experiments using a mouse model showed that DPC-exosome treatment significantly enhanced hair follicle regeneration, increased follicle size, and improved hair density. The findings suggest that DPC-exosomes are critical mediators of hair follicle development and may serve as a potential therapeutic tool for hair loss (Level V).

Rehman and co-workers ^50^ provided a comprehensive review of plant-derived exosomes (PDEs), highlighting their biogenesis, physicochemical characteristics, and therapeutic potential as cross-kingdom nano-regulators. The authors emphasized that PDEs differ substantially from mammalian exosomes in cargo composition, scalability, stability, and immunogenicity; notably, their larger size range (50–200 nm) and enriched lipid, protein, metabolite, and miRNA content support efficient uptake and long-distance intercellular communication across species. Advances in isolation and characterization technologies—including ultracentrifugation, size-exclusion chromatography, asymmetric-flow field-flow fractionation, and PEG-based precipitation—were summarized, as well as the engineering strategies that enable PDEs to serve as biocompatible carriers for nucleic acids, small molecules, and natural bioactives. Importantly, the review highlighted growing evidence that plant miRNAs packaged within PDEs can modulate gene expression in mammalian recipient cells, influencing inflammation, oxidative-stress pathways, immune responses, and metabolic signaling. These mechanisms overlap with key regulatory axes essential for hair follicle cycling—such as Wnt/β-catenin activation, cytokine modulation, melanocyte signaling, and dermal papilla homeostasis—suggesting that PDEs may hold translational relevance for hair regeneration, anti-inflammatory scalp treatments, and pigmentation disorders. Collectively, Rehman et al.’s synthesis positions plant-derived exosomes as promising natural nanocarriers for regenerative, dermatologic, and emerging trichologic applications (Level V).

Chu and co-workers ^51^ presented a comprehensive review of herbal medicine–derived exosome-like nanovesicles (HMDNVs), highlighting their biological characteristics, therapeutic advantages, and emerging relevance in oncology. Although this review focused primarily on cancer, several mechanistic insights are directly translatable to hair biology. The authors emphasized that HMDNVs—lipid bilayer vesicles enriched with plant-specific lipids, proteins, RNAs, and natural metabolites—can modulate inflammation, oxidative stress, macrophage polarization, and Wnt/β-catenin signaling, all of which are central pathways in hair follicle cycling and regeneration. Their discussion of cross-kingdom miRNA transfer is particularly relevant, as plant-derived miRNAs have been shown in other studies to regulate mammalian gene expression associated with cytokine signaling, melanocyte activity, and epithelial homeostasis. These mechanisms parallel those reported in hair-growth studies using ginger-, ginseng-, and *Momordica charantia*–derived nanovesicles, suggesting that HMDNVs may also influence follicular stem-cell activation, dermal papilla function, and pigmentation dynamics. Together, the work of Chu et al. expands the biological rationale for exploring plant-derived nanovesicles as therapeutic candidates not only in oncology but also in emerging trichologic applications such as androgenetic alopecia, alopecia areata, and repigmentation disorders (Level V).

Collectively, these studies indicate encouraging signals across AGA, AA, and CIA with generally favorable safety; however, the clinical evidence remains preliminary and heterogeneous, underscoring the need for adequately powered randomized trials and standardized manufacturing/characterization and dosing protocols.

## Discussion

Exosomes have emerged as a novel therapeutic tool in trichology, offering promising results in various hair loss disorders, including androgenetic alopecia (AGA), alopecia areata (AA), and chemotherapy-induced alopecia (CIA). While much of the evidence is still in the preclinical or early clinical stage, the available literature provides valuable insights into exosomes’ therapeutic potential, mechanisms of action, and limitations. This section discusses the strength and quality of evidence for different hair loss conditions and evaluates the key factors influencing outcomes.

Key regenerative and immunomodulatory mechanisms—including Wnt/β-catenin, PI3K/AKT, VEGF, and TGF-β/SMAD signaling—are summarized schematically in Figure 1 to illustrate the shared molecular pathways across AGA, AA, and CIA.

To preserve clarity, preclinical and clinical findings were synthesized separately, and study designs were appraised qualitatively. While signals are encouraging, the overall strength of human evidence remains limited by small samples, short follow-up, and heterogeneous protocols (see Table 1).

Oxford evidence levels were assigned to facilitate appraisal, revealing that most human studies fall within Levels II–IV, underscoring the need for randomized, controlled investigations.

AGA, the most common form of hair loss, is characterized by progressive hair follicle miniaturization influenced by genetic and hormonal factors, particularly dihydrotestosterone (DHT). Exosome therapy has shown significant promise in addressing AGA, with moderate to strong evidence emerging from preclinical studies and a growing number of clinical trials.

Preclinical studies demonstrate that exosomes derived from mesenchymal stem cells (MSCs), adipose stem cells (ASCs), dermal papilla cells (DPCs) and plant sources activate key signaling pathways critical for follicle regeneration. Liang et al.^40^ reported that adipose mesenchymal stromal cell-derived exosomes carrying miR-122-5p effectively counteracted DHT-induced inhibitory effects on dermal papilla cells by targeting the TGF-β1/SMAD3 signaling pathway. Similarly, studies by Fu et al.^30^ and Mao et al.^34^ highlighted the ability of MSC exosomes to stimulate Wnt/β-catenin and RAS/ERK pathways, resulting in enhanced follicular development and angiogenesis.

Clinical evidence, while limited, is encouraging. Wan et al.^19^ conducted a prospective cohort study in 72 AGA patients, demonstrating significant improvements in hair density, thickness, and follicular cycling with minimal adverse effects. Additionally, Park et al.^42^ reported positive outcomes in a retrospective analysis of 39 AGA patients treated with ADSC-derived exosomes. These findings suggest that exosome therapy is effective in mitigating follicular miniaturization and promoting hair regrowth in AGA. However, the lack of large-scale randomized controlled trials (RCTs) limits the generalizability of these results, necessitating further high-quality studies to confirm efficacy and establish standardized protocols.

Compared with conventional options such as minoxidil, finasteride, or PRP, exosome therapy may provide a broader regenerative effect via multi-pathway modulation. However, given the absence of head-to-head trials, it should currently be regarded as an adjunctive or investigational approach rather than a replacement.

AA is an autoimmune disorder characterized by patchy hair loss caused by immune-mediated destruction of hair follicles. The therapeutic potential of exosomes in AA lies in their immunomodulatory properties, which can suppress inflammation and restore hair follicle cycling.

Preclinical evidence supports the efficacy of exosomes in AA. Hu et al.^35^ demonstrated that umbilical mesenchymal stem cell (uMSC)-derived exosomes enhanced keratinocyte proliferation and migration while reducing inflammatory infiltration around hair follicles in a mouse model of AA. The study attributed these effects to the activation of Wnt/β-catenin and PI3K/AKT pathways, highlighting the role of exosomes in immune regulation and follicular regeneration. Similarly, Li et al.^43^ and Ogawa et al.^47^ reported that exosomes containing specific bioactive molecules, such as miR-218-5p and growth factors, effectively modulated immune responses and promoted hair regrowth in AA models.

Exosomes exert clinically relevant immunomodulation in alopecia areata by reshaping both innate and adaptive responses while supporting follicular immune privilege. Cargoed microRNAs and proteins can attenuate NF-κB and IFN-γ/JAK-STAT signaling, promote regulatory T-cell skewing, and dampen cytotoxic activity at the perifollicular unit, mechanisms consistent with enhanced keratinocyte migration/proliferation and reduced inflammatory infiltration seen in AA models.^35^ Dermal/keratinocyte-derived vesicles also interface with β-catenin/TGF-β pathways implicated in immune-privilege maintenance and follicle cycling.^47^ Translationally, early human signals (case reports/series) suggest potential for regrowth and even pigment restoration in selected patients, but prospective trials should stratify by disease activity/severity, incorporate immune biomarkers (e.g., perifollicular CD8+/Treg ratio, CXCL10), and define dose/interval and delivery to standardize effect size.^40^

Clinical evidence for exosome therapy in AA is limited to case reports and small case series. Bento et al. described a case where a patient with AA achieved complete hair regrowth and restored natural pigmentation after exosome therapy. However, the lack of control groups and small sample sizes in such studies reduce the strength of evidence. Larger, randomized trials are required to confirm the efficacy of exosomes in AA and to identify the optimal dosing and delivery methods.

CIA is a distressing side effect of cancer treatment, resulting from the cytotoxic effects of chemotherapy on rapidly dividing hair follicle cells. Exosomes offer a potential solution by promoting follicular regeneration and mitigating chemotherapy-induced damage.

Preclinical studies provide strong evidence for the efficacy of exosomes in CIA. Wu et al.^46^ demonstrated that exosomes derived from dermal papilla cells and adipose stem cells enhance hair follicle proliferation, reduce apoptosis, and improve vascularization in animal models. These effects were attributed to the activation of Wnt/β-catenin and VEGF signaling pathways, which are critical for follicular repair and angiogenesis.

Clinical evidence for exosome therapy in CIA remains sparse but promising. Norooznezhad et al.^39^ reported a case of persistent CIA successfully treated with MSC-derived exosome-enriched extracellular vesicles, resulting in significant hair regrowth and scalp coverage. The study highlighted the regenerative properties of exosomes and their ability to deliver bioactive molecules, such as growth factors and cytokines, to damaged follicles. While these findings are encouraging, the lack of large-scale clinical studies limits the strength of evidence, underscoring the need for further research.

Emerging evidence suggests that combining exosome therapy with other modalities, such as low-level laser therapy (LLLT) or platelet-rich plasma (PRP), may have synergistic effects. Mirzadeh et al.^36^ reported significant improvements in hair density and thickness in AGA patients treated with a combination of LLLT and autologous exosome therapy, compared to either treatment alone. Similarly, Wang et al.^38^ demonstrated enhanced hair follicle regeneration when exosome therapy was augmented with lactotransferrin (LTF), a protein known for its anti-inflammatory properties.

These findings indicate that combination therapies can enhance the efficacy of exosome-based treatments by targeting multiple pathways involved in hair follicle regeneration. However, more studies are needed to identify the most effective combinations and to optimize treatment protocols.

The growing body of evidence highlights the therapeutic potential of exosomes in trichology. Preclinical studies provide robust mechanistic insights, demonstrating the ability of exosomes to activate key signaling pathways, enhance cellular proliferation, and modulate immune responses. Clinical studies, while limited, show promising results in improving hair density, thickness, and scalp health across various hair loss conditions.

In routine practice, exosome interventions carry several practical limitations that warrant explicit counseling and documentation. Products are heterogeneous across source cells, isolation methods, potency assays, and storage conditions, and many formulations lack approval as medicinal products in key jurisdictions, creating regulatory uncertainty. To ensure clinical reliability, GMP-compliant manufacture, defined particle characterization, sterility/mycoplasma testing, and a batch-level certificate of analysis (COA) should be required before patient use.

Quality and safety depend on GMP-grade manufacture with batch certificates of analysis, endotoxin thresholds, and sterility/mycoplasma testing; absent these, contamination risk and variable particle counts/cargo can undermine reproducibility. Although serious adverse events appear rare, immunogenicity and theoretical oncogenicity remain insufficiently characterized long term; avoid treatment in active malignancy, autoimmune flare, or infected/dermatitic scalp. Protocols also vary (dose, interval, injection vs. topical delivery, and combinations), limiting comparability and making standardized maintenance schedules difficult to define. In addition, several limitations exist. The majority of studies are preclinical, with limited translation to human trials. Small sample sizes, short follow-up periods, and the lack of standardized protocols reduce the generalizability of findings. Additionally, variability in exosome isolation and characterization methods poses challenges to reproducibility and clinical implementation.

**Figure 2:**
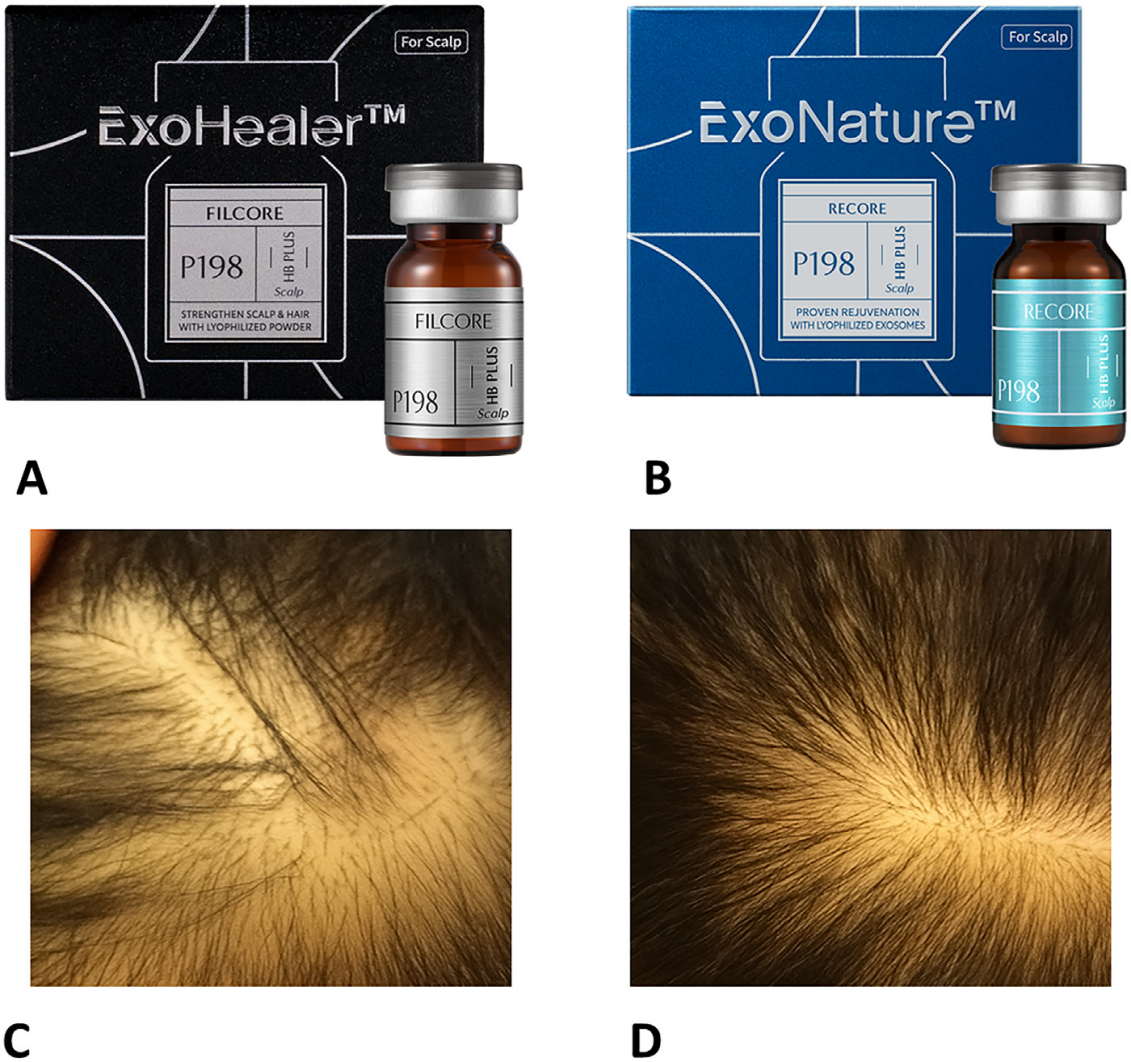
Exosome-based scalp formulations and example clinical use. (A) P198 ExoHealer FILCORE HB PLUS (Primoris International Co., Ltd., Korea), a hair-specific formulation containing human umbilical cord blood stem cell–derived exosomes for scalp regeneration. (B) P198 ExoNature RECORE HB PLUS (Primoris International Co., Ltd., Korea), a Panax ginseng–derived exosome preparation designed for hair and scalp rejuvenation. (C) Baseline clinical photograph of a patient with diffuse scalp thinning before P198 ExoNature RECORE HB PLUS treatment. (D) Same patient after five sessions of P198 ExoNature RECORE HB PLUS applied topically to the scalp and then delivered with 0.5‑mm microneedling at 3‑week intervals, demonstrating visible improvement in hair density and scalp coverage.

However, many companies that develop products with exosomes as the main ingredient are conducting research and development to overcome various shortcomings of exosome raw materials. For example, Primoris International has launched a product that can be moved and stored at room temperature after freeze-drying raw materials containing exosomes to powder them in order to maintain the stability of exosomes (Figure 2).

Future research should prioritize multicenter randomized controlled trials with unified endpoints (e.g., trichoscopic density, hair shaft diameter) and standardized dosing intervals. Development of consensus guidelines for exosome isolation, potency assays, and delivery routes will be crucial for translating these findings into routine dermatologic and aesthetic practice.

## Conclusion

Exosomes represent a transformative approach in trichology, offering a minimally invasive and biologically active solution for hair loss disorders. Strong preclinical evidence supports their efficacy in promoting follicular regeneration, while emerging clinical data demonstrate promising results in AGA, AA, and CIA.

However, most available human data derive from case reports or small cohort studies (Levels II–IV), with heterogeneous methodology and short follow-up durations. The lack of standardized outcome measures, variable exosome purity, and inconsistent dosing intervals currently limit generalizability.

Despite the current limitations, including the lack of large-scale RCTs and standardized protocols, exosomes hold significant potential as a next-generation therapeutic tool in hair restoration. Continued research and clinical trials will be pivotal in unlocking their full potential and ensuring their successful translation into routine clinical practice. Future clinical translation will also require harmonized regulatory frameworks and GMP-compliant manufacturing with batch-level quality control to ensure reproducibility and patient safety.

## Data availability statement

The data is available by requesting to corresponding author.

## Author contributions

All authors have reviewed and approved the article for submission. Conceptualization, Kyuho Yi. Writing – Original Draft Preparation, Kar Wai Alvin Lee; Jong Keun Song; Sa Rang Lee. Writing – Review & Editing, Han Earl Lee; Olena Sydorchuk; Sa Rang Lee; Eunwoo Yu; Sea Hwan Ki; Tae-Hyun Ki; Han Ah Reum Son; Seung Yong Shi; You-kyoung Cho; Han Earl Le; Arash Jalali; Kyu-Ho Yi. Visualization, Kyu-Ho Yi; Arash Jalali; Supervision, Kyu-Ho Yi.

## Funding

There is no financial disclosure to report.

## Ethical approval

Not required.

## Declaration of competing interest

I acknowledge that I have considered the conflict of interest statement included in the “Author Guidelines.” I hereby certify that, to the best of my knowledge, that no aspect of my current personal or professional situation might reasonably be expected to significantly affect my views on the subject I am presenting.

## References

[1] Otberg N., Finner A.M., Shapiro J. (2007). Androgenetic alopecia. Endocrinol Metab Clin North Am.

[2] Pratt C.H., King L.E., Messenger A.G., Christiano A.M., Sundberg J.P. (2017). Alopecia areata. Nat Rev Dis Primers.

[3] Chon S.Y., Champion R.W., Geddes E.R., Rashid R.M. (2012). Chemotherapy-induced alopecia. J Am Acad Dermatol.

[4] Olsen E.A., Weiner M.S., Delong E.R., Pinnell S.R. (1985). Topical minoxidil in early male pattern baldness. J Am Acad Dermatol.

[5] Gupta A.K., Talukder M., Williams G. (2022). Comparison of oral minoxidil, finasteride, and dutasteride for treating androgenetic alopecia. J Dermatol Treat.

[6] Stout S.M., Stumpf J.L. (2010). Finasteride treatment of hair loss in women. Ann Pharmacother.

[7] Badran K.W., Sand J.P. (2018). Platelet-rich plasma for hair loss: review of methods and results. Facial Plastic Surg Clin.

[8] Jimenez F., Alam M., Vogel J.E., Avram M. (2021). Hair transplantation: basic overview. J Am Acad Dermatol.

[9] Ghorbani R., Hosseinzadeh S., Azari A. (2024). The current status and future direction of extracellular nano-vesicles in the alleviation of skin disorders. Curr Stem Cell Res Ther.

[10] Xie M., Wu D., Li G., Yang J., Zhang Y.S. (2021). Exosomes targeted towards applications in regenerative medicine. Nano Select.

[11] Kalluri R., LeBleu V.S. (2020). The biology, function, and biomedical applications of exosomes. Science.

[12] Liu F., Liu S., Luo X. (2024). Combatting ageing in dermal papilla cells and promoting hair follicle regeneration using exosomes from human hair follicle dermal sheath cup cells. Exp Dermatol.

[13] Hong P., Yang H., Wu Y., Li K., Tang Z. (2019). The functions and clinical application potential of exosomes derived from adipose mesenchymal stem cells: a comprehensive review. Stem Cell Res Ther.

[14] Papadopoulos K.S., Piperi C., Korkolopoulou P. (2024). Clinical applications of adipose-derived stem cell (ADSC) exosomes in tissue regeneration. Int J Mol Sci.

[15] Vyas K.S., Kaufman J., Munavalli G.S., Robertson K., Behfar A., Wyles S.P. (2023). Exosomes: the latest in regenerative aesthetics. Regen Med.

[16] Liang Y., Tang X., Zhang X., Cao C., Yu M., Wan M. (2023). Adipose mesenchymal stromal cell-derived exosomes carrying MiR-122-5p antagonize the inhibitory effect of dihydrotestosterone on hair follicles by targeting the TGF-β1/SMAD3 signaling pathway. Int J Mol Sci.

[17] Gupta A.K., Wang T., Rapaport J.A. (2023). Systematic review of exosome treatment in hair restoration: preliminary evidence, safety, and future directions. J Cosmet Dermatol.

[18] Mirzadeh S., Hashesmi T., Jafari A.M. (2024). Combination of low-level laser therapy and autologous exosome therapy in hair growth; case series. Sch J Med Case Rep.

[19] Wan J., Kim S.B., Cartier H. (2025). A prospective study of exosome therapy for androgenetic alopecia. Aesthetic Plast Surg.

[20] Park B.S., Choi H.I., Huh G., Kim W.S. (2022). Effects of exosome from adipose-derived stem cell on hair loss: a retrospective analysis of 39 patients. J Cosmet Dermatol.

[21] Amini F., Teh J.J., Tan C.K., Tan E.S.S., Ng E.S.C. (2025). A pilot randomized controlled trial (RCT) evaluating the efficacy of an exosome-containing plant extract formulation for treating male alopecia. Life.

[22] Lee E., Choi M.S., Cho B.S. (2024). The efficacy of adipose stem cell-derived exosomes in hair regeneration based on a preclinical and clinical study. Int J Dermatol.

[23] Ersan M., Ozer E., Akin O., Tasli P.N., Sahin F. (2024). Effectiveness of exosome treatment in androgenetic alopecia: outcomes of a prospective study. Aesthetic Plast Surg.

[24] Norooznezhad A.H., Yarani R., Payandeh M. (2023). Treatment of persistent chemotherapy-induced hair loss (Alopecia) with human mesenchymal stromal cells exosome enriched extracellular vesicles: a case report. Heliyon.

[25] Hassan L., Samin K.A., Mohsin S., Asif M.I., Maheshwary N., Ahmed A. (2024). Compare the efficacy of PRP intervention VS exosomes for hair loss, a case series study. Dermis.

[26] Chen X., Pang J., Li J. (2025). Mesenchymal stem cell exosomes therapy for acquired trichorrhexis nodosa: a case series. J Cosmet Dermatol.

[27] Queen D., Avram M.R. (2025). Exosomes for treating hair loss: a review of clinical studies. Dermatol Surg.

[28] Schaffer S., Tehrani L., Koechle B. (2025). A scoping review of exosome delivery applications in hair loss. Cureus.

[29] Chu S., Jia L., Li Y. (2025). Exosome-derived long non-coding RNA AC010789.1 modified by FTO and hnRNPA2B1 accelerates growth of hair follicle stem cells against androgen alopecia by activating S100A8/wnt/β-catenin signalling. Clin Transl Med.

[30] Fu Y., Han Y.T., Xie J.L. (2025). Mesenchymal stem cell exosomes enhance the development of hair follicle to ameliorate androgenetic alopecia. World J Stem Cells.

[31] Lu C., Ding Y., Zhang R. (2025). Platelet-rich plasma-derived exosomes stimulate hair follicle growth through activation of the Wnt/β-catenin signaling pathway. Regen Ther.

[32] Norouzi F., Aghajani S., Vosoughi N. (2024). Exosomes derived stem cells as a modern therapeutic approach for skin rejuvenation and hair regrowth. Regen Ther.

[33] Zhou Y., Seo J., Tu S., Nanmo A., Kageyama T., Fukuda J. (2024). Exosomes for hair growth and regeneration. J Biosci Bioeng.

[34] Mao Y., Liu P., Wei J. (2024). Exosomes derived from umbilical cord mesenchymal stem cell promote hair regrowth in C57BL6 mice through upregulation of the RAS/ERK signaling pathway. J Transl Internal Med.

[35] Hu S., Zhang J., Ji Q. (2024). Exosomes derived from uMSCs promote hair regrowth in alopecia areata through accelerating human hair follicular keratinocyte proliferation and migration. Cell Biol Int.

[36] Cheng M., Ma C., Chen H.D., Wu Y., Xu X.G. (2024). The roles of exosomes in regulating hair follicle growth. Clin Cosmet Investig Dermatol.

[37] Wang G., Wang Z., Zhang J. (2023). Treatment of androgenetic alopecia by exosomes secreted from hair papilla cells and the intervention effect of LTF. J Cosmet Dermatol.

[38] Gupta A.K., Hall D.C., Rapaport J.A., Paradise C.R. (2023). Exosomes and hair restoration. Adv Cosmet Surg.

[39] Kost Y., Muskat A., Mhaimeed N., Nazarian R.S., Kobets K. (2022). Exosome therapy in hair regeneration: a literature review of the evidence, challenges, and future opportunities. J Cosmet Dermatol.

[40] Li Y., Wang G., Wang Q., Zhang Y., Cui L., Huang X. (2022). Exosomes secreted from adipose-derived stem cells are a potential treatment agent for immune-mediated alopecia. J Immunol Res.

[41] Zhang Y., Su J., Ma K., Li H., Fu X., Zhang C. (2022). Photobiomodulation promotes hair regeneration in injured skin by enhancing migration and exosome secretion of dermal papilla cells. Wound Repair Regen.

[42] Kim H., Jang Y., Kim E.H. (2022). Potential of colostrum-derived exosomes for promoting hair regeneration through the transition from telogen to anagen phase. Front Cell Dev Biol.

[43] Wu J., Yang Q., Wu S. (2021). Adipose-derived stem cell exosomes promoted hair regeneration. Tissue Eng Regen Med.

[44] Ogawa M., Udono M., Teruya K., Uehara N., Katakura Y. (2021). Exosomes derived from fisetin-treated keratinocytes mediate hair growth promotion. Nutrients.

[45] Nilforoushzadeh M.A., Aghdami N., Taghiabadi E. (2021). Effects of adipose-derived stem cells and platelet-rich plasma exosomes on the inductivity of hair dermal papilla cells. Cell J (Yakhteh).

[46] Ajit A., Nair M.D., Venugopal B. (2021). Exploring the potential of mesenchymal stem cell–derived exosomes for the treatment of alopecia. Regen Eng Transl Med.

[47] Hu S., Li Z., Lutz H. (2020). Dermal exosomes containing miR-218-5p promote hair regeneration by regulating β-catenin signaling. Sci Adv.

[48] Kwack M.H., Seo C.H., Gangadaran P. (2019). Exosomes derived from human dermal papilla cells promote hair growth in cultured human hair follicles and augment the hair-inductive capacity of cultured dermal papilla spheres. Exp Dermatol.

[49] Zhou L., Wang H., Jing J., Yu L., Wu X., Lu Z. (2018). Regulation of hair follicle development by exosomes derived from dermal papilla cells. Biochem Biophys Res Commun.

[50] Rehman T.U., Li H., Martuscelli M. (2025). Plant-derived exosomes: Nano-inducers of cross-kingdom regulations. Pharmaceuticals.

[51] Chu K., Liu J., Zhang X. (2024). Herbal medicine-derived exosome-like nanovesicles: A rising star in cancer therapy. Int J Nanomed.

